# Lung Ultrasound in the Diagnosis of COVID-19 Pneumonia: Not Always and Not Only What Is COVID-19 “Glitters”

**DOI:** 10.3389/fmed.2021.707602

**Published:** 2021-07-19

**Authors:** Carla Maria Irene Quarato, Antonio Mirijello, Michele Maria Maggi, Cristina Borelli, Raffaele Russo, Donato Lacedonia, Maria Pia Foschino Barbaro, Giulia Scioscia, Pasquale Tondo, Gaetano Rea, Annalisa Simeone, Beatrice Feragalli, Valentina Massa, Antonio Greco, Salvatore De Cosmo, Marco Sperandeo

**Affiliations:** ^1^Institute of Respiratory Diseases, COVID-19 Center, Policlinico Universitario “Riuniti” di Foggia, Foggia, Italy; ^2^Department of Medical and Surgical Sciences, University of Foggia, Foggia, Italy; ^3^Department of Internal Medicine, COVID-19 Unit, Istituto di Ricovero e Cura a Carattere Scientifico (IRCCS) Fondazione Casa Sollievo della Sofferenza, Foggia, Italy; ^4^Department of Emergency Medicine and Critical Care, Emergency Medicine Unit, COVID-19 Center, Istituto di Ricovero e Cura a Carattere Scientifico (IRCCS) Fondazione Casa Sollievo Della Sofferenza, Foggia, Italy; ^5^Department of Radiology, Istituto di Ricovero e Cura a Carattere Scientifico (IRCCS) Casa Sollievo della Sofferenza, Foggia, Italy; ^6^Department of Emergency Medicine and Critical Care, Intensive Care Unit, COVID-19 Center, Istituto di Ricovero e Cura a Carattere Scientifico (IRCCS) Fondazione Casa Sollievo Della Sofferenza, Foggia, Italy; ^7^Department of Radiology, “Vincenzo Monaldi” Hospital-Association of periOperative Registered Nurses (AORN) Ospedale Dei Colli, Naples, Italy; ^8^Department of Medical, Oral and Biotechnological Sciences - Radiology Unit “G. D'Annunzio, ” University of Chieti-Pescara, Chieti, Italy; ^9^Department of Medical Sciences, Geriatric and COVID-19 Unit, Istituto di Ricovero e Cura a Carattere Scientifico (IRCCS) Fondazione Casa Sollievo della Sofferenza, Foggia, Italy; ^10^Department of Medical Sciences, Unit of Interventional and Diagnostic Ultrasound of Internal Medicine, Istituto di Ricovero e Cura a Carattere Scientifico (IRCCS) Fondazione Casa Sollievo della Sofferenza, Foggia, Italy; ^11^Diagnostic and Interventional Lung Ultrasonology at the Bachelor in Medicine and Surgery and the Postgraduate School of Respiratory Disease, University of Foggia, Foggia, Italy

**Keywords:** lung ultrasound, computed tomography, COVID-19, comorbidities, screening method, sensitivity, specificity

## Abstract

**Background:** In the current coronavirus disease-2019 (COVID-19) pandemic, lung ultrasound (LUS) has been extensively employed to evaluate lung involvement and proposed as a useful screening tool for early diagnosis in the emergency department (ED), prehospitalization triage, and treatment monitoring of COVID-19 pneumonia. However, the actual effectiveness of LUS in characterizing lung involvement in COVID-19 is still unclear. Our aim was to evaluate LUS diagnostic performance in assessing or ruling out COVID-19 pneumonia when compared with chest CT (gold standard) in a population of SARS-CoV-2-infected patients.

**Methods:** A total of 260 consecutive RT-PCR confirmed SARS-CoV-2-infected patients were included in the study. All the patients underwent both chest CT scan and concurrent LUS at admission, within the first 6–12 h of hospital stay.

**Results:** Chest CT scan was considered positive when showing a “typical” or “indeterminate” pattern for COVID-19, according to the RSNA classification system. Disease prevalence for COVID-19 pneumonia was 90.77%. LUS demonstrated a sensitivity of 56.78% in detecting lung alteration. The concordance rate for the assessment of abnormalities by both methods increased in the case of peripheral distribution and middle-lower lung location of lesions and in cases of more severe lung involvement. A total of nine patients had a “false-positive” LUS examination. Alternative diagnosis included chronic heart disease (six cases), bronchiectasis (two cases), and subpleural emphysema (one case). LUS specificity was 62.50%. Collateral findings indicative of overlapping conditions at chest CT were recorded also in patients with COVID-19 pneumonia and appeared distributed with increasing frequency passing from the group with mild disease (17 cases) to that with severe disease (40 cases).

**Conclusions:** LUS does not seem to be an adequate tool for screening purposes in the ED, due to the risk of missing some lesions and/or to underestimate the actual extent of the disease. Furthermore, the not specificity of LUS implies the possibility to erroneously classify pre-existing or overlapping conditions as COVID-19 pneumonia. It seems more safe to integrate a positive LUS examination with clinical, epidemiological, laboratory, and radiologic findings to suggest a “virosis.” Viral testing confirmation is always required.

## Introduction

With the global spread of the novel severe acute respiratory syndrome coronavirus 2 (SARS-CoV-2), the coronavirus disease-2019 (COVID-19) has currently become a major health problem worldwide. Although COVID-19 can involve several organs and apparatus, it mainly represents a respiratory disease affecting pulmonary parenchyma. Therefore, an extensive literature discussion on the use of chest imaging for the diagnosis and follow-up of this disease is still ongoing.

Portable bedside Chest X-ray (CXR) is a valid tool for the evolutionary monitoring of pneumonia (1). However, CXR is less sensitive than CT in revealing the typical GGOs in the early stage of disease (2, 3). A large number of studies tried to describe chest CT characteristics of hospitalized patients with COVID-19 pneumonia (4–6). However, in the first 3 days from the onset of clinical symptoms, chest CT may not show lung alterations (7). In addition, it should be underlined that CT patterns are not completely specific because other diseases or comorbidities (e.g., heart failure, other viral or bacterial pneumonia, and chronic pulmonary diseases) can give similar findings (8).

A too high demand for exams, often useless, together with the frequent need for CT sanitization negatively impacts on the functionality of radiology services. For these reasons, the American College of Radiology (ACR) has rigorously and strongly expressed against the use of chest CT as a first-line diagnostic screening for COVID-19 pneumonia, suggesting the use of portable CXR, when possible, to minimize the risk of SARS-CoV-2 viral shedding (9). Chest CT should be used sparingly and reserved for hospitalized, symptomatic patients with specific clinical indications (e.g., to rule out comorbidities or complications). At present, a reverse-transcription polymerase chain reaction (RT-PCR) testing from nasopharyngeal swab remains the gold standard for diagnosis confirmation.

Lung ultrasound (LUS) is a mobile, fast, repeatable, and non-invasive technology that does not expose the patient to radiation. Ultrasound machines are widely available, quick to clean, and easily transportable bedside, thus avoiding the movement of patients through hospital and to expose other health workers to the risk of infection. However, actual available information on the diagnostic value of LUS in COVID-19 pneumonia, especially in comparison with chest CT findings, is still not sufficiently clear.

On this background, the present study aimed to evaluate the performance of LUS examination as a screening tool for assessing signs of pneumonia in a population of confirmed SARS-CoV-2-infected patients presenting to the emergency department (ED). Chest CT was regarded as the gold standard reference method.

## Materials and Methods

### Study Design

This is a prospective single-center observational study aiming to evaluate the diagnostic performance of LUS in assessing or ruling out COVID-19 pneumonia, compared with chest CT as standard reference. The study received the ethical approval from the local ethical committee (COVID-19-CSS, n. 46/2020) and was carried out according to the principles of the Declaration of Helsinki.

Inclusion criteria were (1) a written informed consent for all the procedures signed by participants or their legal guardians; (2) age >18 years old; (3) presence of suggestive symptomatology: fever, cough, sore throat, dyspnea, diarrhea, myasthenia, ageusia, and anosmia; and (4) a positive result from SARS-CoV-2-specific RT-PCR on nasopharyngeal swabs collected at admission.

From the beginning of the COVID-19 emergency until December 28, 2020, a total of 1,012,689 RT-PCR tests for SARS-CoV2 were carried out in the Apulia region, among which 87,084 positive cases emerged (8.60% of the total sample) (10). Starting from these data, we calculated that, at a significant type I error rate of 5% and a 95% confidence interval (CI), a minimum of 121 patients was required to create a representative sample size.

Between March and October 2020, we enrolled a cohort of 260 consecutive symptomatic patients with a confirmed SARS-CoV-2 infection admitted to our Research Institute “Fondazione Casa Sollievo della Sofferenza,” San Giovanni Rotondo, Italy. This guaranteed us an appropriate sample size for the study. All the enrolled patients underwent to chest CT scan and concurrent LUS examination during the first 6–12 h of hospital stay from admission. Unavailability of the CT and/or LUS assessment within this range of time was considered an exclusion criterion.

Reporting of the study was guided by the consolidated Standards for Reporting Diagnostic Accuracy Studies (STARD) recommendations (11).

### Clinical Evaluation

Nasopharyngeal swabs were collected from each patient at admission, as per guidelines (12); all the enrolled patients had a RT-PCR-confirmed infection. The following clinical data were recorded: medical history (demographic data, comorbidities, symptoms, and date of their onset), physical examination (body temperature, blood pressure, heart rate, respiratory rate, and oxygen saturation), and laboratory test results. Patients were classified according to the best respiratory supportive option to maintain an acceptable peripheral saturation (SpO_2_) >93% (i.e., spontaneous breathing, conventional oxygen therapy, high-flow nasal cannula, continuous positive airway pressure or bi-level positive airway pressure, need for intubation) and consequently allocated in the different COVID-19 units of our hospital.

### Chest CT

Chest CT examination was performed using a multidetector CT scanner with 64 channels. The detailed parameters for CT acquisition were as follows: tube voltage, 120 kVp; tube current, standard (reference mAs, 60–120); slice thickness, 0.5 mm; and reconstruction interval, 0.3–1.0 mm. All CT images were acquired at full inspiration (impossible in a few severely ill patients) with the patient in the supine position and without contrast medium. Cleaning and disinfection procedures of CT scan followed each exam, requiring ~20 min per patient. Chest CT scans were interpreted by a radiologist with 32 years of experience in thoracic imaging and reviewed by a second expert in thoracic imaging to reach a consensus. We considered positive patients with typical or indeterminate chest CT pattern for COVID-19, as defined by the RSNA classification system (13). Presence of the following lung lesions, categorized according to the *Fleischner Society: Glossary of Terms for Thoracic Imaging* (14), was recorded: ground-glass opacities (GGOs) (i.e., hazy areas of increased attenuation without obscuration of the underlying vascular markings); crazy-paving pattern (i.e., scattered or diffuse ground-glass attenuation with superimposed interlobular septal thickening and intralobular lines); and consolidations (i.e., parenchymal opacities obscuring underlying vessels). CT patterns were subsequently graded as mild disease (i.e., focal and sporadic GGOs), moderate disease (i.e., crazy-paving pattern), and severe disease (i.e., pulmonary consolidations).

The distribution of lesions was classified with regard to the central lung (i.e., the inner two-third of the lung tissue) and peripheral lung (i.e., the outer one-third of the lung). Unilateral or bilateral involvement was also specified. The location of lung abnormalities was noted and classified by dividing the lungs into an upper middle-zone and a middle-lower zone. The upper-middle zone was defined as the portion of lungs above a transversal plane passing through the hila and the middle-lower zone as the portion of lungs below such transversal plane.

In addition, we also evaluated presence of collateral findings not typically associated to COVID-19 pneumonia and involving airways (i.e., bronchiectasis), pulmonary parenchyma (i.e., nodules, emphysema), pulmonary interstitium (i.e., reticulation, nodularity, and honeycombing), heart and vessels (i.e., cardiomegaly, pulmonary artery caliber, artery to bronchus ratio), pleura (i.e., thickening, nodularity, and pleural effusion), and pericardium (i.e., pericardial effusion).

### Lung Ultrasound

LUS examination was performed with an Esaote MyLab-25 GOLD or a My-LabTwice scanner (Esaote-Biomedica, Genoa, Italy) using a multifrequency convex probe (3–5 MHz and 3–8 MHz) and an adequate setting for the adult thoracic study (gain: max 50%, focus pointed on the hyperechoic pleural line, activation of the tissue harmonic). Patients were examined in a sitting or semisitting position; in the case of critical patients, supine and lateral positions were used. The assessment covered the entire area of each lung, from the base up to the ipsilateral apex, with longitudinal and transversal scans along the anatomical transversal demarcation lines of the chest wall. The anterior scans were made following the parasternal, mid-clavicular, and anterior-axillary lines; the lateral scans were made following the mid-axillary and posterior-axillary lines, and the posterior scans were made following the mid-scapular and paravertebral lines.

LUS exams were performed and interpreted by three expert sonographers, with 10–32 years of experience in diagnostic and interventional ultrasound, that were blinded to chest CT scan results. Each sonographer was dressed in full personal protection equipment (PPE) during examination. The approximate duration of the entire LUS examination was 15 min. Videoclips were recorded for each patient and later blindly re-examined by another sonographer with 20 years of experience in lung ultrasound in order to reach consensus.

The following LUS findings were assessed: normal lung; thickness and appearance of the hyperechoic pleural line; presence/absence of B-lines; presence/absence of consolidations; presence/absence of subpleural nodules; and presence/absence of pleural effusion.

An ultrasound pattern consisting in a thin, regular, and continuous hyperechoic pleural line (i.e., the hyperechoic line viewable at the interface between soft tissues of the chest wall and the aired lung surface) followed by horizontal, equally spaced “A-lines” (i.e., echogenic reverberation artifacts produced by bouncing of echo between the pleural line and probe) was regarded as a sign of normal aired lung (15).

A conventional cut-off of 3.0 mm was used to define the pleural line as normal (≤ 3.0 mm) or thickened (>3.0 mm) (16). Pleural line's abnormalities were noted if it appeared irregularly thickened (irregularity), showed focal interruptions (fragmented), or presented less definite contours (blurred) (16, 17).

B-lines were defined as continuous and parallel hyperechoic artifacts, arising from the pleural line and extending indefinitely along the direction of the US beam on the screen (vertical artifacts) (15). Well-spaced B-lines in a number <3 were regarded as a normal finding; coalescent or not B lines in a number ≥3 between two ribs in a single scan were considered a positive sign of disease.

Consolidations were defined as subpleural large hypoechoic or liver-like areas interrupting the overlying pleural line echogenicity and characteristically showing blurred deep margins (18).

Subpleural nodules were defined as subpleural hypoechoic small lesions (<3 mm), round or oval in shape, interrupting the hyperechoic pleural line (16).

Pleural effusion was defined as an anechoic free fluid collection in the pleural space showing a dependent location or a posterior location with the patient in the supine position (15).

The chest was divided in an upper-middle zone and a middle-lower zone by a horizontal circumferential line passing through the nipples, and location of each LUS finding was recorded.

### Statistical Analysis

Numerical variables were presented as mean values ± standard deviation (SD); categorical variables were presented as counts and percentages. Considering chest CT as the “gold standard” method, we estimated sensitivity, specificity, positive likelihood ratio, negative likelihood ratio, positive predictive value, and negative predictive value of LUS in assessing COVID-19 lesions with a 95% CI. The empiric receiver operating characteristic (ROC) curve analysis was used to study the diagnostic performance of LUS vs. chest CT in discriminating positive cases of COVID-19 pneumonia from negative ones. We defined area under the ROC curve (AUC) values of 0.50–0.59, 0.60–0.69, 0.70–0.79, and ≥0.80 as none, poor, acceptable, and excellent discrimination, respectively. The concordance rate between chest CT scan and LUS examination was defined as the number of concordant results over the total number of cases assessed. On the basis of chest CT findings, our cohort of COVID-19 patients was divided into three groups with different degree of pneumonia severity. The concordance rate was examined in each group. Analyses were performed with GraphPad Prism 6.0.

## Results

### General Findings

A total of 260 consecutive patients (139 males, 53% and 121 females, 47%) who met the inclusion criteria were enrolled in the study. The mean ± SD age at admission was 69 ± 12 years (range, 20–100), and 64% of patients were ≥65 years of age. The 25% of patients had a BMI ≥30 kg/m^2^, with a mean ± SD BMI of 27 ± 3 kg/m^2^ (range, 18–46). More than half of the patients (59%) showed cardiovascular comorbidities, with hypertension being the most prevalent (47%). Diabetes and chronic respiratory diseases were observed in 26 and 15% of the cases, respectively. Chronic kidney disease and past or present neoplasm accounted for the 12 and 8% of the study sample.

The mean interval from symptoms onset until admission was 5 ± 1 days. A total of 186 patients were admitted to COVID-19 emergency department for observation. Among them, 99 patients were on spontaneous breathing and 89 required a conventional oxygen therapy. Fifty-four patients, of which 25 required high-flow nasal cannula (HFNC), and 29 continuous positive airway pressure (CPAP), were admitted to a COVID-19 ward. A total of 15 patients (five on CPAP and 13 requiring BiPAP) were admitted to a COVID-19 subintensive or intensive care unit. Three of them were immediately intubated due to sudden respiratory worsening.

Table 1 summarizes the baseline clinical characteristics of patients at admission.

**Table 1:** Demographic, clinical, and laboratory data of the 260 patients included in the study at admission.

**Demographic characteristics**	
Age (mean ± SD)	69 ± 12 (20–100)
Sex, male (*n*, %)	139 (53%)
Sex, female (*n*, %)	121 (47%)
BMI [kg/m^2^ (mean ± SD)]	27 ± 3 (18–46)
Smoking habits, current (*n*, %)	16 (6%)
Smoking habits, former (*n*, %)	38 (15%)
Smoking habits, not assessed (*n*, %)	13 (5%)
**Comorbidities**	
Hypertension (*n*, %)	121 (47%)
Diabetes (*n*, %)	67 (26%)
Chronic respiratory disease (*n*, %)	44 (17%)
Cardiovascular disease (*n*, %)	42 (16%)
Chronic kidney disease (*n*, %)	31 (12%)
Anamnestic neoplasm (*n*, %)	20 (8%)
Autoimmune disorders (*n*, %)	14 (5%)
**Symptoms**	
Fever (*n*, %)	170 (65%)
Cough (*n*, %)	141 (54%)
Dyspnea (*n*, %)	137 (53%)
Sore thoat (*n*, %)	80 (31%)
Myasthenia (*n*, %)	64 (25%)
Gastrointestinal symptoms (*n*, %)	35 (13%)
Anosmia (*n*, %)	28 (11%)
Ageusia	18 (7%)
Chest tightness (*n*, %)	5 (2%)
Onset of symptoms [days (mean ± SD)]	5 ± 2 (1–14)
**Physical examination**	
Body temperature [^°^C (mean ± SD)]	37.2 ± 0.9 (36.2–40.0)
SBP [mmHg (mean ± SD)]	126 ± 15 (60–200)
DBP [mmHg (mean ± SD)]	74 ± 9 (40–120)
Hearth rate [bpm (mean ± SD)]	91 ± 15 (38–195)
Respiratory rate (mean ± SD)	28 ± 9 (12–50)
SpO_2_ [% (mean ± SD)]	92 ± 3 (70–100)
**Laboratory test**	
WBC × 10^9^/L	8.79 ± 4.74 (0.47–86.6)
CRP (mg/dl)	21.74 ± 19.36 (0.2–101.0)
PCT (μg/L)	0.77 ± 2.01 (0.02–29.07)
Creatinine (mg/dl)	1.12 ± 0.55 (0.2–8.5)
GFR (ml/min)	87.38 ± 32.59 (5.0–250.0)
NT-ProBNP (pg/ml)	480 ± 30 (120–1,840)
D-dimer (ng/ml)	2,413 ± 2,662 (30–67,145)
**Respiratory support required**	
Spontaneous breathing	99 (38%)
COT	89 (34%)
HFNC	25 (10%)
CPAP	35 (13%)
NIV	9 (4%)
Intubation	3 (1%)

*BMI, body mass index; SBP, systolic blood pressure; DBP, diastolic blood pressure; SpO_2_, peripheral oxygen saturation; WBC, white blood cells; CRP, C-reactive protein; PCT, procalcitonin; GFR, glomerular filtration rate; COT, conventional oxygen therapy; HFNC, high-flow nasal cannula; CPAP, continuous positive airway pressure; NIV, non-invasive ventilation*.

A total of 236 patients (91%) showed COVID-19–related lung abnormalities at chest CT scan, while 24 patients (9%) had a negative chest CT scan. Concurrent LUS examination resulted positive in 143 patients (55%). Among them, 134 patients had signs of COVID-19 pneumonia at chest CT scan (“true-positive” LUS examination) while nine patients did not (“false-positive” LUS examination).

**Figure 1:**
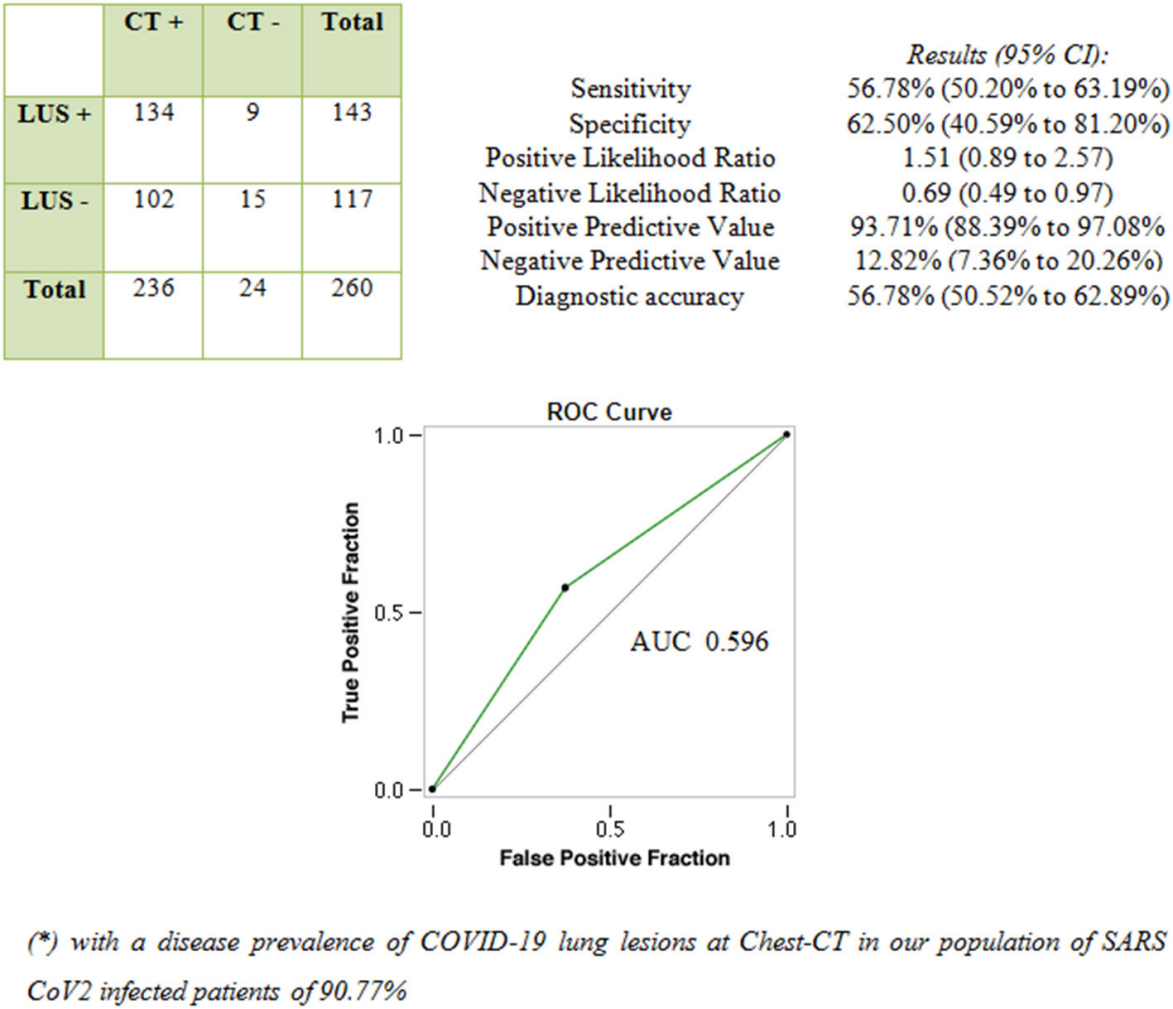
Performance of LUS in diagnosing COVID-19 pneumonia compared with chest CT scan (“gold standard” test).

Of the 117 patients (45%) with negative LUS examination, 15 were “true negatives” while 102 were “false negatives.” In these cases, LUS was not able to detect COVID-19–related pulmonary abnormalities, as shown by chest CT. Figure 1 shows the performance of LUS for the diagnosis of COVID-19 pneumonia considering chest CT as “gold standard.”

The concordance rate between LUS examination and CT scan in assessing lesions was higher for peripheral distribution and middle-lower zone location. In other words, LUS was not able to detect lesions located in the central lung (Table 2).

**Table 2:** Concordance rate between LUS and CT scan in assessing lung lesions according to their distribution and location.

	**Chest CT**	**LUS+**	**LUS–**	**Concordance rate**
**Lesions distribution**				
Central distribution	12	0	12	0%
Peripheral distribution	145	110	35	76%
Diffuse distribution	79	24	55	30%
**Lesions location**				
Upper-middle zone	114	21	93	18%
Middle-lower zone	249	134	115	54%

**Figure 2:**
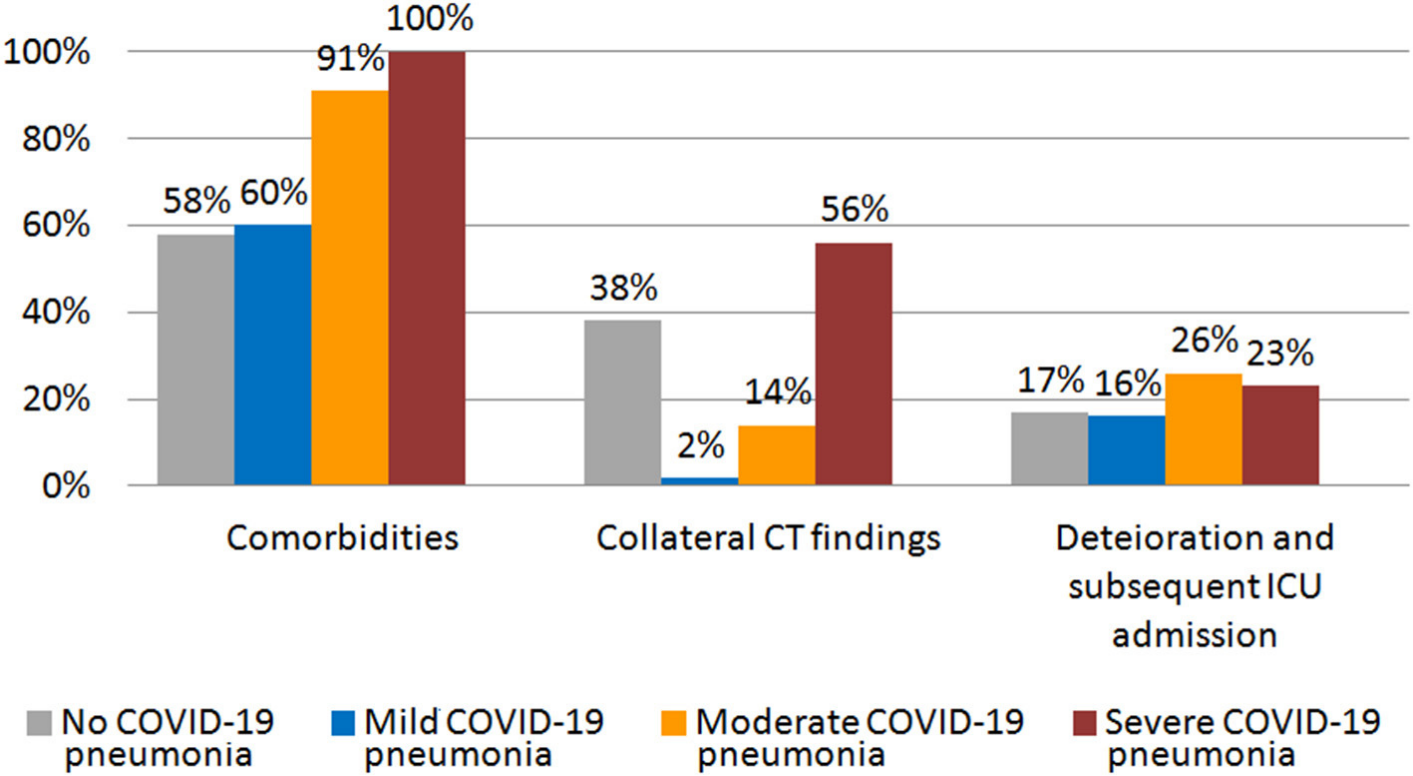
Rate of comorbidities, collateral CT findings, and percentage of patients that showed worsening and required subsequent ICU admission in different groups of initial CT scan severity.

Our cohort was divided in four groups based on the severity of lung alterations, as shown at CT scan. Figure 2 shows the rate of comorbidities, collateral findings on chest CT, and the proportion of patients requiring subsequent ICU admission in the four groups of patients.

### Mild COVID-19 Pneumonia

A total of 43 patients (16.5%) had a mild radiological pneumonia characterized by focal and sporadic ground-glass opacities (GGOs) at chest CT scan. This pattern was observed in patients with less-severe clinical presentations (mean respiratory rate: 14 ± 1; mean SpO_2_: 96 ± 2%). The mean ± SD age was 59 ± 11 years and 35% of patients were ≥65 years of age. Twenty-six percent of patients had a BMI of ≥30 kg/m^2^. The 60% of patients showed comorbidities, with hypertension being the most prevalent (51%). Diabetes and chronic respiratory diseases were observed in 21 and 16% of the patients, respectively. Chronic heart failure was recorded in the 7% of the patients.

At admission, 37 patients (86%) were in spontaneous breathing and six (14%) on conventional oxygen therapy. The mean interval from symptoms onset until admission for these patients was 4 ± 1 days.

**Figure 3:**
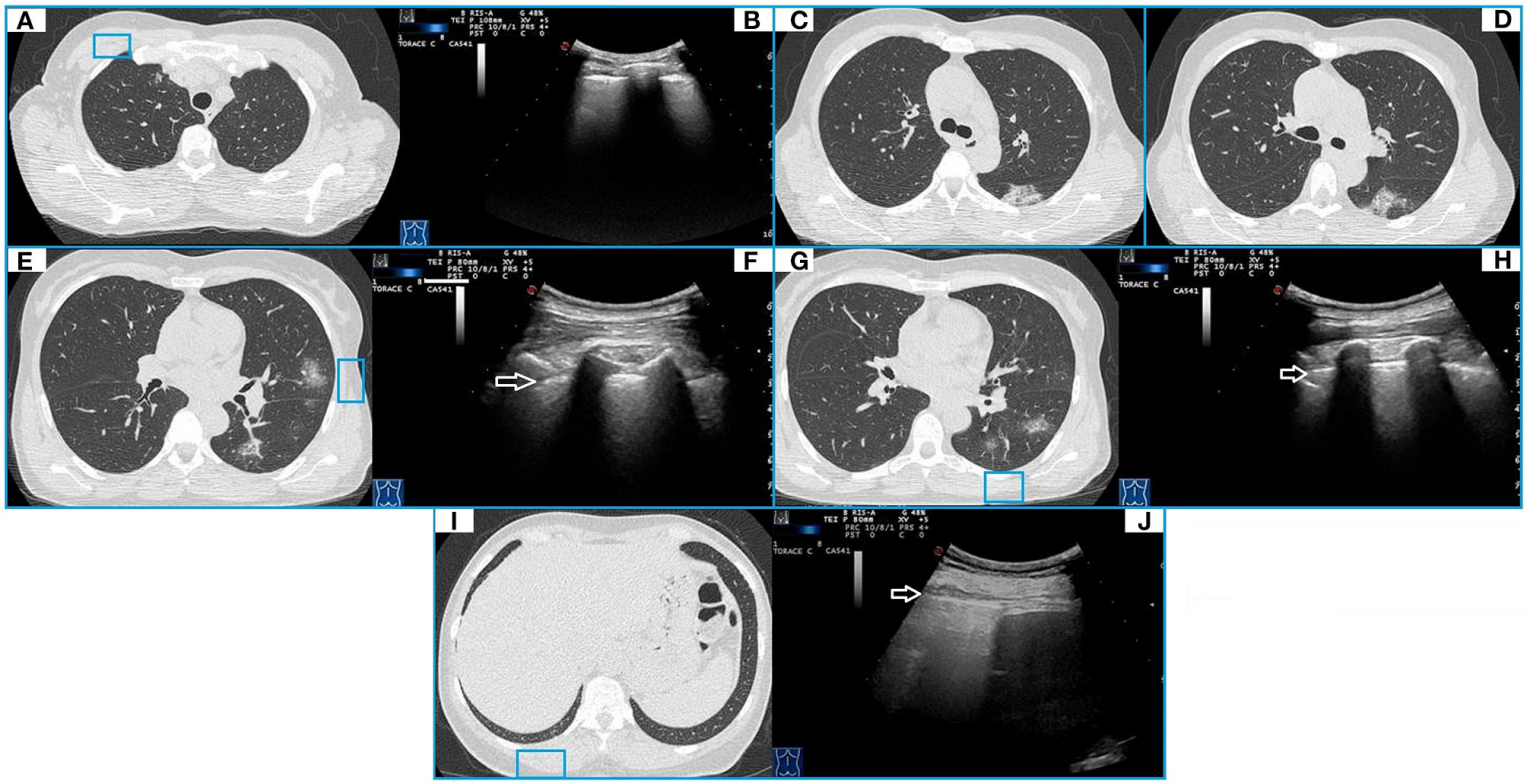
CT and TUS findings in mild COVID-19 pneumonia. A 57-year-old female patient presenting in ED with a 1 week fever, dyspnea, cough, and fatigue. The RT-PCR assay on nasopharyngeal swab confirmed the suspect for COVID-19 pneumonia. In **(A)**, axial CT scan passing through the upper lobes shows a peripheral focal ground glass opacity located in the anterior parenchyma of right upper lobe and not adhering to the pleural surface. In **(B)**, ultrasound scan with a convex probe (6 MHz) and thoracic setting in the upper region of the chest [blue box in **(A)**] shows a normal hyperechoic pleural line (white arrow) without B-lines. Axial CT scans passing through the tracheal carina [in **(C)**] and the middle lung regions [in **(D)**] show mixed areas of ground glass/consolidation with subpleural distribution in the left lower lobe. These lesions are located in the retroscapular area, resulting in being not visible on ultrasound scans. Axial CT scans passing through the middle lung regions [in **(E)**] and the lower lobes [in **(G)**] show rounded focal ground glass opacities with central distribution in the lingula and the left lower lobe. In **(F,H)**, ultrasound scans with a convex probe (6 MHz) and thoracic setting, corresponding to the blue boxes in the respective CT scans, show a normal hyperechoic pleural line without B-lines (white arrow). In **(I)**, axial CT scan passing through the basal lung regions shows no significant lesions. In **(J)**, ultrasound scan with a convex probe (6 MHz) and thoracic setting, corresponding to the blue box in the respective CT scan, shows a normal hyperechoic pleural line (white arrow) near the correspondent right posterior costofrenic sinus.

Of the 43 patients with mild disease, LUS examination was not able to demonstrate any evidence of pulmonary disease in 36 patients (84%), whose lesions did not reach the pleural surface. On the contrary, in the remaining seven patients (16%) showing bilateral GGOs reaching the pleural surface, LUS showed a mild irregular and thickened hyperechoic pleural line, followed by >3 focal B-lines. In three of them (7%), LUS showed also subpleural nodulations interrupting the continuity of the hyperecoic pleural line. Concurrent LUS findings were bilateral in five of seven patients (71%) (Figure 3).

In one patient, a hypoechoic subpleural nodulation measuring ~6 mm at US was associated with a pulmonary nodule in the lower right lobe reaching the pleural surface and measuring 8 mm in diameter at chest CT (Table 3).

**Table 3:** Concordance rate between LUS and CT in patients with initial mild CT scan findings.

**Mild pneumonia**			
Chest CT	LUS		
Focal and sporadic ground-glass opacities (GGOs)	Thickened and irregular hyperechoic pleural + >3 focal B-lines		*Concordance rate*
43	Yes	No	16%
	7	36	
Focal and sporadic ground-glass opacities (GGOs)	Hypoechoic subpleural nodules		*Concordance rate*
43	Yes	No	7%
	3	40	
*Collateral findings at chest CT*		*LUS findings*	
Subpleural nodule of 8 mm (*n* = 1)		Hypoechoic subpleural nodulation measuring ~6 mm	

A total of seven patients (16%) with initial mild CT scan findings required ICU admission because of subsequent deterioration.

### Moderate COVID-19 Pneumonia

A total of 122 patients (47%) had a moderate disease, as suggested by a radiological crazy-paving pattern consisting in patchy or extensive peripheral GGOs, completely or incompletely adherent to pleura, associated with smooth interlobular and intralobular septal thickening. Patients in this group presented with a mean respiratory rate of 30 ± 5 and a mean SpO_2_ of 92 ± 3%. The mean ± SD age was 68 ± 9 years, and 65% of patients were ≥65 years of age. Twenty-sex percent of patients had a BMI of ≥30 kg/m^2^. The 91% of the patients showed comorbidities. Diabetes and chronic respiratory diseases and chronic heart failure were observed in 25, 17, and 13% of the patients, respectively.

At admission, 44 patients (36%) were in spontaneous breathing, 31 (25%) required a conventional oxygen therapy, 19 (16%) were on HFNC, 24 (20%) required a ventilatory support by CPAP, three (2%) needed a support by non-invasive ventilation (NIV) and one (1%) required intubation. The mean interval from symptoms onset until diagnosis was 6 ± 1 days.

**Figure 4:**
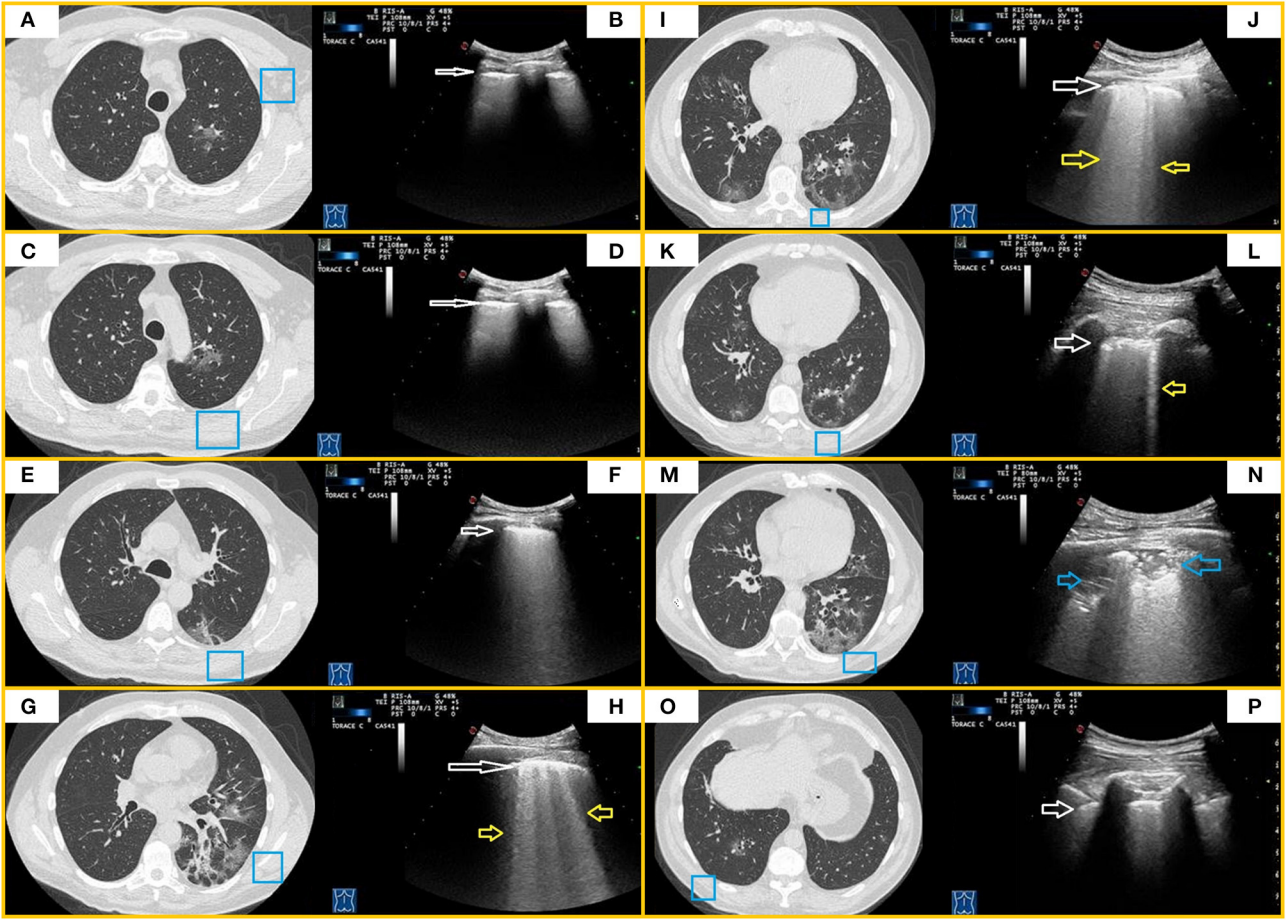
CT and TUS findings in moderate COVID-19 pneumonia. A 67-year-old male patient presenting in ED with fever and cough for 10 days. The RT-PCR assay on nasopharyngeal swab confirmed the suspect for COVID-19 pneumonia. In **(A,C)**, axial CT scans passing through the upper lobes show central areas of pure ground glass in the left upper lobe. In **(E)**, axial CT scan passing through the carina level shows a peripheral area of pure ground glass in the left superior lower lobe. In **(B,D,F)**, ultrasound scans with a convex probe (6 MHz) and thoracic setting, corresponding to the blue boxes on the respective CT scans, show a thickened hyperechoic pleural line without B-lines (white arrow). In **(G,I,K)**, axial CT scans passing through middle-lower zones show multiple confluent areas of ground glass opacities, with peri-bronchovascular and subpleural distribution, in the lingula and right and left lower lobes. Associated linear opacities with peri-lobular pattern and bronchiectasis are observable in left lower lobes. Initial bronchiectasis is visible in the middle lobe. In **(H,J,L)**, ultrasound scans with a convex probe (6 MHz) and thoracic setting, corresponding to the blue boxes in the respective CT scans, show an irregular and thickened hyperechoic pleural line (white arrow) with focal or coalescent B-lines (yellow arrows). In **(M)**, axial CT scan passing through the basal lung regions shows multiple subpleural patchy areas of pure ground glass opacities adherent to pleural surface in the posterior region of the left lower lobe. In **(N)**, ultrasound scan with a convex probe (6 MHz) and thoracic setting, corresponding to the blue box in the respective CT scan, shows mixed hypoechoic subpleural irregular consolidation (blue arrow). In **(O)**, axial CT scan passing through the basal lung regions shows some pure ground glass opacities, the largest of which is localized in the right lower lobe along the bronchovascular structures. In **(P)**, ultrasound scan with a convex probe (6 MHz) and thoracic setting, corresponding to the blue box in the respective CT scans, shows a thickened hyperechoic pleural line (white arrow) without B-lines.

**Figure 5:**
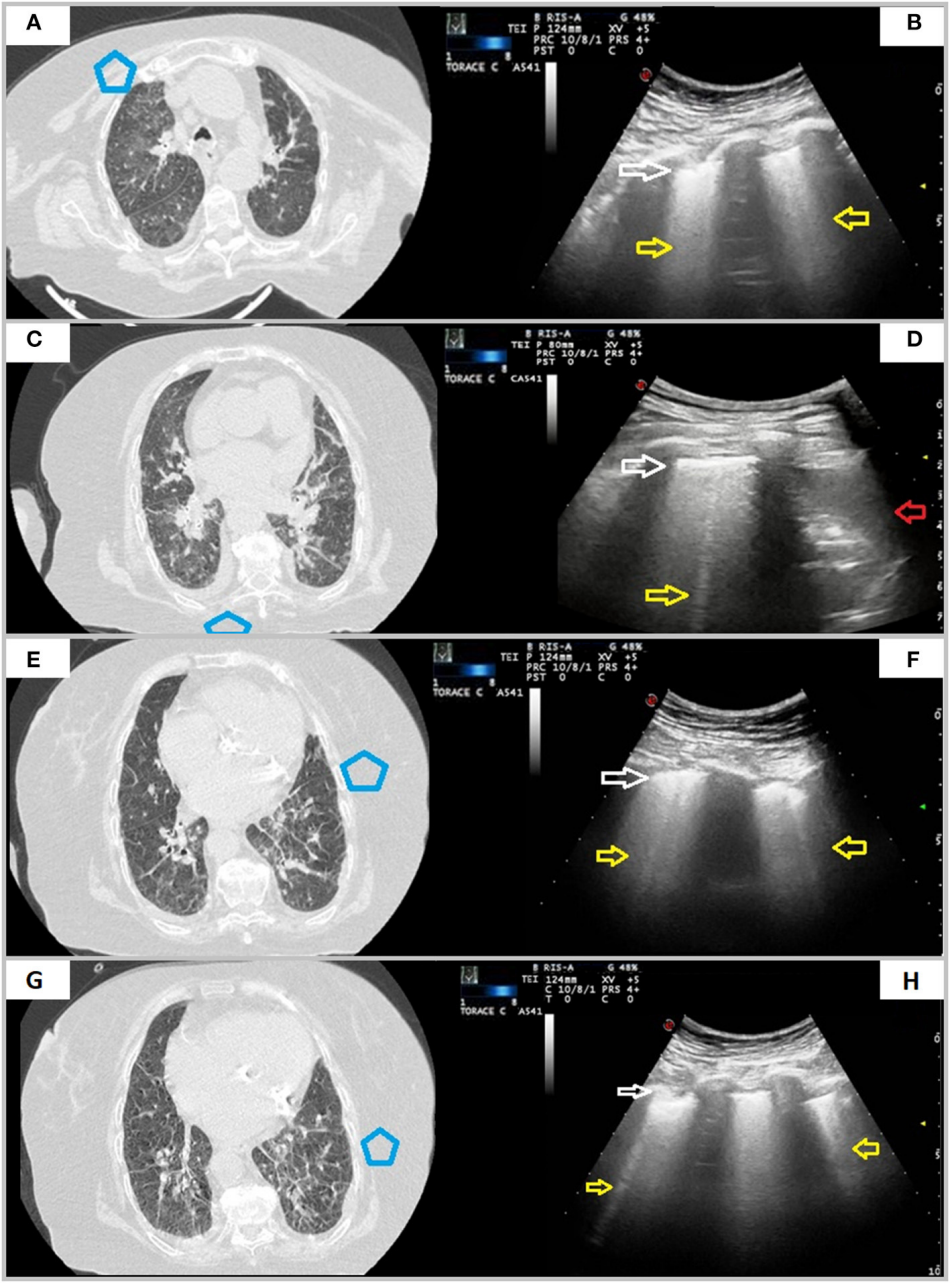
A case of overlapping cardiogenic pulmonary edema. A 76-year-old female patient presenting in ED with dyspnea for 6 days. The RT-PCR assay on nasopharyngeal swab resulted positive for SARS-CoV-2 infection. In **(A,C,E,G)**, axial CT scans reveal smooth interlobular septal thickening, fissural thickening, mixed GGO and consolidation with central distribution, increased artery to bronchus ratio, cardiomegaly, and bilateral pleural effusion. In **(B,D,F,H)**, ultrasound scans with a convex probe (6 MHz) and thoracic setting, corresponding to the blue boxes in the respective CT scans, show an irregular and thickened hyperechoic pleural line (white arrow) followed by focal or coalescent B-lines (yellow arrows). Right posterior ultrasound scan in **(H)** also underlines presence of pleural effusion (red arrow).

In this group, LUS examination resulted positive in 56 out of 122 patients (46%). Reported findings were an irregular, thickened, and blurred hyperechoic pleural line with B-lines, focal or confluent, below it. LUS showed also subpleural nodulations in 34 patients (29%) and mixed hypoechoic subpleural irregular consolidation in 18 patients (15%). These findings were associated to confluent supleural areas of ground glass on chest CT. Chest CT findings were bilateral in 116 patients, while LUS showed a bilateral pattern in 36 patients (31%) (Figure 4). Three patients showed a pleural effusion both at chest CT and concurrent LUS, while LUS detected a minimal pleural effusions in other two patients. In five patients, chest CT scan also showed overlapping signs of heart failure, including a smooth peribronchovascular interstitium thickening, cardiomegaly, enlarged main pulmonary artery, increased artery to bronchus ratio, and pericardial effusion (Figure 5). In addition, five patients had lung fibrosis (one with honeycombing and four with a reticular pattern), three bronchiectasis, and four subpleural emphysema (Table 4).

**Table 4:** Concordance rate between LUS and CT in patients with initial moderate CT scan findings.

**Moderate pneumonia**			
Chest CT	LUS		
Crazy-paving pattern	Thickened, irregular and blurred hyperechoic pleural + focal or confluent B-lines		*Concordance rate*
122	Yes	No	46%
	56	66	
Crazy-paving pattern	Hypoechoic subpleural nodules		*Concordance rate*
122	Yes	No	28%
	34	88	
Crazy-paving pattern	Hypo-/echoic consolidations with ill-defined margins and mixed hyper-/hypoechoic spot within		*Concordance rate*
122	Yes	No	15%
	18	104	
Pleural effusion	Pleural effusion		*Concordance rate*
3	Yes	No	100%
	5	0	
*Collateral findings at chest CT*		*LUS findings*	
Heart failure (*n* = 5)		Thickened and blurred hyperechoic pleural + confluent B-lines + pleural effusion	
Lung fibrosis (*n* = 5) Bronchiectasis (*n* = 3) Subpleural emphysema (n = 4)		Thickened, irregular and blurred hyperechoic pleural + focal or confluent B-lines	

Thirty-two patients (26%) with initial moderate CT scan findings required ICU admission because of subsequent deterioration.

### Severe COVID-19 Pneumonia

A total of 71 patients showed chest CT scan findings consisting with severe disease (e.g., peripheral dense pulmonary consolidations). These findings were bilateral in the majority of patients (66/71 cases). Patients in this group had a clinically severe disease (mean respiratory rate: 39 ± 6; mean SpO_2_: 91 ± 4).

At admission, 48 patients were on conventional oxygen, six were on HFNC, 11 needed a ventilatory support by CPAP, and six by NIV; two patients suddenly worsen and required immediate intubation. The mean interval from symptom onset until admission was 7 ± 2 days. The mean ± SD age was 72 ± 11 years and 73% of patients were ≥65 years of age. Twenty-eight percent of patients had a BMI of ≥30 kg/m^2^. All patients (100%) showed comorbidities. Diabetes, chronic heart failure, and chronic respiratory diseases were observed in 30, 25, and 15% of the patients, respectively.

**Figure 6:**
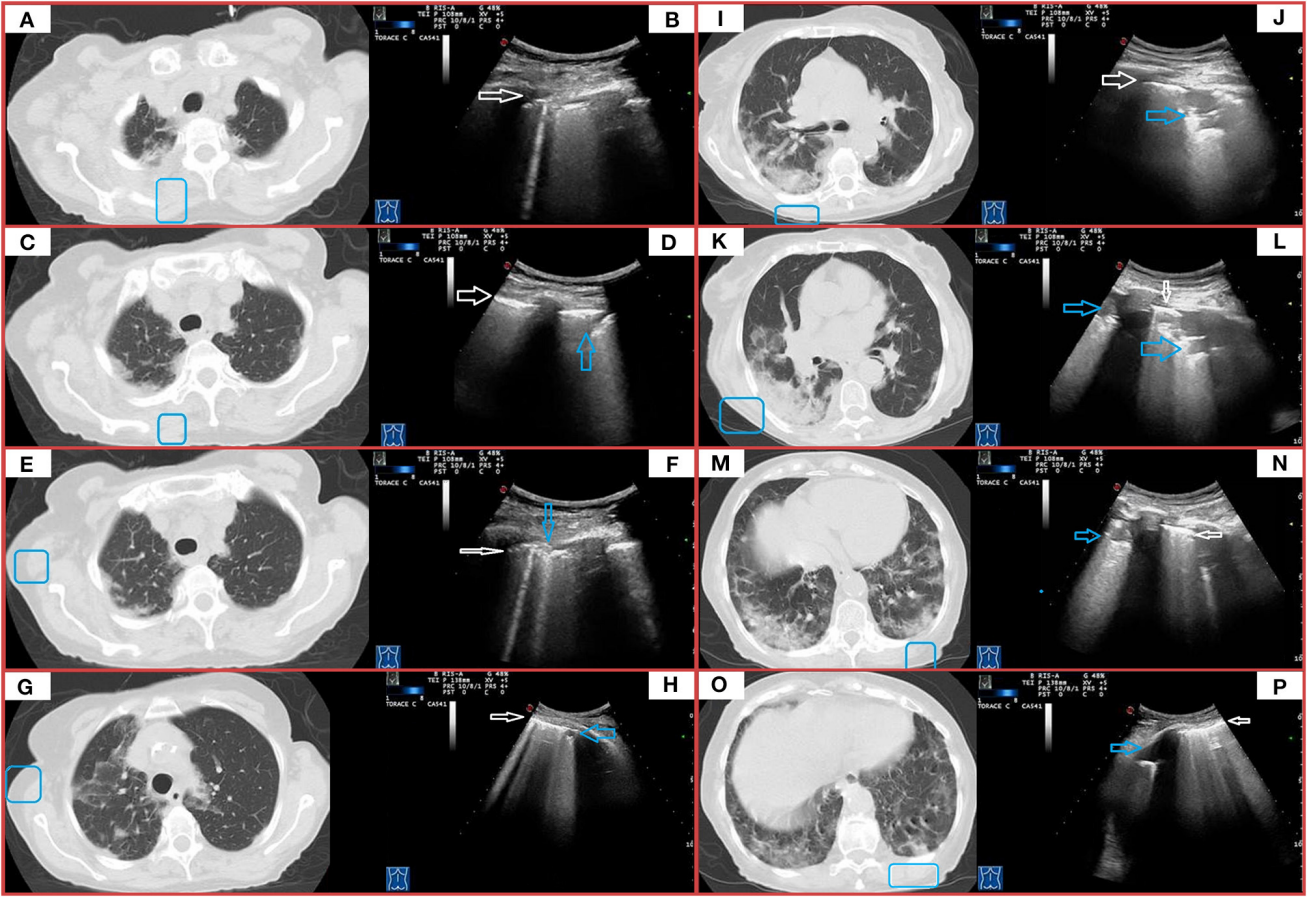
CT and TUS findings in severe COVID-19 pneumonia. A 72-year-old male patient presenting in ED with 1 week fever, dyspnea, cough, and fatigue. The RT-PCR assay on nasopharyngeal swab confirmed the suspect for COVID-19 pneumonia. In **(A)**, axial scan passing through the apical segments of right and left upper lobes shows a mixed pattern of ground glass opacities and minimal consolidations with subpleural and posterior distribution extending in the retroscapular and retrocostovertebral areas. In **(B)**, ultrasound scan with a convex probe (6 MHz) and thoracic setting, corresponding to the blue box in the respective CT scan, allows to see an irregular pleural line (white arrow) with a single B-line (corresponding the US-accessible portion of the lesion). In **(C)**, axial scan passing through the right and left upper lobes shows patchy subpleural ground glass lesions, also extending in the retroscapular and retrocostovertebral areas, mostly on the right side. In **(D)**, ultrasound scan with a convex probe (6 MHz) and thoracic setting, corresponding to the blue box in the respective CT scan, shows a thickened hyperechoic pleural line (white arrow) with a mixed hypoanechoic subpleural irregular nodulation (blue arrow), corresponding to the US-accessible portion of these patchy subpleural ground glass lesions. In **(E)**, axial CT scan passing through the upper lobes shows patchy subpleural ground glass opacities, with prevalent posterior distribution and extending also in the retroscapular areas in the right upper lobe and a focal ground glass opacity localized in the subpleural parenchyma of the paramediastinal region in the right upper lobe. In **(F)**, ultrasound scan with a convex probe (6 MHz) and thoracic setting, corresponding to the blue box in the respective CT scan, shows a thickened hyperechoic pleural line (white arrow) with two B-line and a focal subpleural hyperechoic micronodulation (blue arrow) corresponding to the US-accessible part of the retroscapular lesion. In **(G)**, axial CT scans passing through the upper lobes shows patchy and diffuse areas of ground glass opacities, with a minimal involvement of the retroscapular area, in the right upper lobe. In the left upper lobe, a mixed area of ground glass and consolidation is visible in the subpleural parenchyma of paramediastinal region. In **(H)**, ultrasound scan with a convex probe (6 MHz) and thoracic setting, corresponding to the blue box in the respective CT scans, shows a thickened hyperechoic pleural line (white arrow) with coalescent B-lines and a subpleural hypoechoic micronodulation, corresponding the US-accessible part of these patchy and diffuse areas of GGO. In **(I)**, axial CT scan passing through the level of the carina shows subpleural mixed consolidation and ground glass lesions in the apical segment of the right lower lobe and the lateral regions of the right upper lobe. On the left side, minimal subpleural ground glass opacities are present in the apical segment of the lower lobe. In **(K,M,O)**, axial CT scans passing through the middle-lower region show a large and confluent consolidation mixed with ground glass opacities, mostly located in the posterior regions of the middle and right lower lobes. Both lower lobes show multiple confluent areas of ground glass opacities in a predominant subpleural and posterior distribution, associated with minimal consolidations. In **(J,L,N,P)**, ultrasound scans with a convex probe (6 MHz) and thoracic setting, corresponding to the blue boxes in the respective CT scans, show a thickened hyperechoic pleural line (white arrow) with mixed hypoechoic subpleural consolidations (blue arrows), representing the portion of these consolidations completely adhering to the pleural surface.

In this group, LUS examination resulted positive in 100% of patients. In 69 of them (97%), LUS identified hypoechoic consolidations with ill-defined margins and mixed hyper-/hypoechoic spot within. In 65 patients (92%), LUS imaged also subpleural nodulations interrupting the continuity of pleural lines. In the remaining two patients (3%), LUS showed only a blurred, irregular, and thickened hyperechoic pleural line with confluent B-lines below it. This happened because consolidations showed at chest CT scans were not adherent to the pleural surface. Furthermore, in seven patients (11%) with bilateral consolidations at chest CT, LUS detected consolidations only in one unilateral zone, while in 48 patients (70%), the extension of consolidations was different between the two techniques (Figure 6). A total of 15 patients showed a pleural effusion both at chest CT and LUS. Furthermore, LUS detected a small pleural effusions in the other six patients. Regarding collateral findings, 16 patients in this subgroup had signs of heart failure at CT scan, 15 had bronchiectasis, five received a diagnosis of overlapping bacterial pneumonia with a positive PCT (>2 ng), three had a known history of respiratory disease with subpleural emphysema and one had lung fibrosis with honeycombing (Table 5).

**Table 5:** Concordance rate between LUS and CT in patients with initial severe CT scan findings.

**Severe pneumonia**			
Chest CT	LUS		
Peripheral dense pulmonary consolidations	Hypo-/echoic consolidations with ill-defined margins and mixed hyper-/hypoechoic spot within		*Concordance rate*
71	Yes	No	97%
	69	2	
Peripheral dense pulmonary consolidations	Thickened, irregular, and blurred hyperechoic pleural + focal or confluent B-lines		*Concordance rate*
71	Yes	No	100%
	71	0	
Peripheral dense pulmonary consolidations	Hypoechoic subpleural nodules		*Concordance rate*
71	Yes	No	92%
	65	6	
Pleural effusion	Pleural effusion		*Concordance rate*
15	Yes	No	100%
	21	0	
*Collateral findings at chest CT*		*LUS findings*	
Signs of heart failure (*n* = 16)		Thickened and blurred hyperechoic pleural + confluent B-lines + pleural effusion	
Overlapping bacterial pneumonia (*n* = 5)		Hypo-/echoic consolidations with ill-defined margins and mixed hyper-/hypoechoic spot within	
Bronchiectasis (*n* = 15)		Thickened and irregular hyperechoic pleural + >3 focal B-lines	
Subpleural emphysema (n = 3)			
Lung fibrosis (*n* = 1)			

A total of 16 patients (23%) with initial severe CT scan findings required ICU admission because of subsequent deterioration.

### Patients Without COVID-19 Pneumonia

A total of 24 patients (9%) had a normal chest CT scan, indicating that they had not yet developed signs of pulmonary involvement detectable at CT scan. Patients in this group presented with a mean respiratory rate of 17 ± 4 and a mean SpO_2_ of 94 ± 2%. The mean ± SD age was 68 ± 17 years and 63% of patients were ≥65 years of age. Four percent of patients had a BMI of ≥30 kg/m^2^. Most of the patients (58%) showed comorbidities, with hypertension being the most prevalent (58%). Diabetes and chronic respiratory diseases were observed in 25 and 21% of the patients, respectively. Twenty-five percent of the patients had a known history of chronic heart disease.

The mean interval from symptom onset until diagnosis was 4 ± 2 days. Of these patients, nine (38%) had a “false-positive” LUS examination. In particular, six patients showed a blurred and thickened hyperechoic pleural line with confluent B-lines below it and a mild pleural effusion. Such patients showed only a cardiomegaly on chest CT within a known history of chronic heart disease. The remaining three patients showed a thickened and more irregular pleural line followed by >3 focal B-lines. Of them, two patients had peripheral bronchiectasis and one had subpleural emphysema on chest CT within a known history of COPD (Table 6).

**Table 6:** Concordance rate between LUS and CT scan in patients with a CT scan negative for COVID-19 lung abnormalities.

**Negative for pneumonia**			
Chest CT	LUS		
Negative for COVID-19 lung abnormalities	False-positive LUS	True-negative LUS	*Concordance rate*
24	9	15	63%
*Collateral findings at chest CT*		*LUS findings*	
Cardiomegaly (*n* = 6)		Thickened and blurred hyperechoic pleural + confluent B-lines + pleural effusion	
Bronchiectasis (*n* = 2)		Thickened and irregular hyperechoic pleural + > 3 focal B-lines	
Subpleural emphysema (*n* = 1)		Thickened and irregular hyperechoic pleural + >3 focal B-lines	

At admission, 20 patients (83%) were in spontaneous breathing and four (17%) required a conventional oxygen therapy. Given the absence of signs of pneumonia on CT, respiratory failure in these patients was related to concomitant comorbidities. Four patients (17%) with initial negative CT scan findings required ICU admission because of subsequent deterioration.

## Discussion

In the present emergency condition due to the pandemic spread of SARS-CoV-2, finding an imaging method that allows a rapid and reliable screening of the population for lung involvement can help to avoid, in affected patients, the progression of disease and the delay of hospitalization. Our study highlighted that LUS examination does not represent the most suitable method to meet these needs.

Early reports on LUS performance in COVID-19 diagnosis suggested a comparable or even superior sensitivity to chest CT in detecting lung lesions (19–21). However, the inclusion of few patients and the absence of a systematic comparison between LUS and CT performances do not allow to draw confident conclusions. Available studies evaluating the correlation between LUS findings and CT showed widely heterogeneous results, with an estimated sensitivity for LUS ranging from 15.6 to 100% (22–27). Data deriving from our preliminary experience have shown a low sensitivity of LUS in diagnosing COVID-19 pneumonia (28).

In the present study, LUS showed a sensitivity of 56.8% and a negative predictive value of 12.8% in assessing signs of COVID-19 pneumonia compared with chest CT. These results can be explained by technical limitations inherent in the exploration of aired lung with ultrasound, given that more than 96% of the ultrasound beam is reflected at chest wall tissues/aired lung interface, and ultrasound is not able to image normal pulmonary parenchyma (29). The result of this change of tissue impedance is the production of sonographic artifacts such as the “hyperecoic pleural lines,” “A-lines,” and sporadic “B-lines” (15). Consequently, compared with volumetric chest CT, LUS can be effectively used for detecting only lesions or conditions involving or facing to the superficial pleura. In these circumstances, the acoustic mismatch between the lung parenchyma and chest wall tissues is lowered and the acoustic window on the lung becomes partially or completely open, depending on the degree of loss of aeration. Conversely, as shown also in the present experience, LUS does not allow to explore the central and perihilar regions of the lung and the areas facing to the mediastinal pleura. Lesions not adhering to the parietal pleura, even due to a few millimeters or microns of air, cannot be identified by LUS. Furthermore, due to the anatomic hindrance of bony structures of the thoracic cage, LUS can examine, at best and with the patient in a sitting position, only 70% of the pleural surface (15). These limits have been recently underlined in an interesting review on the role of imaging in COVID-19 pneumonia (30).

Another possible explanation for the lower sensitivity showed by LUS in our study compared with the other ones may lie in the fact that the first wave of COVID-19 pandemic in Southern Italy, specifically in the Apulia region, was not characterized by a number of infections as high as those recorded in Northern Italy or in other European countries. This lower prevalence allowed the access to ERs and consequent hospitalization of many patients even a few days after the onset of symptoms. Consequently, we found a high number of patients with early lung abnormalities that hardly reached the pleura, resulting in being undetectable by LUS. In line with this speculation, the concordance rate between chest CT and LUS in the detection of parenchyma abnormalities increased more the degree of radiological severity of pneumonia. In particular, as GGOs converged and crowded in lung periphery on chest CT passing from a mild to moderate pneumonia, LUS findings ranged from a thickening of the pleural line with underlying discrete or focal B-lines to small subpleural nodulations and areas of consolidation interrupting the continuity of pleural line, finally turning into the frank consolidations of severe pneumonia that were documented on both chest CT and LUS. Nevertheless, the number and extension of lesions identified at chest CT and those identified at US were not exactly the same, neither in more severe cases. This happened because some consolidations or parts of them were located in areas of the lung that were not accessible to ultrasound (e.g., the retroscapular area, the subpleural mediastinal area, or the costovertebral junction regions) or were not completely adherent to the pleural surface. In addition, an excess of subcutaneous fat tissue, as occurs in obese people who are more frequently affected by severe COVID-19 pneumonia, may prevent optimal US imaging (31). As a result, the use of LUS carries a risk of missing some lesions and/or to underestimate the actual extent of the disease. Considering the high possibility of subsequent clinical worsening and need for admission to ICU even in cases with initial mild CT scan findings (16% of cases of mild pneumonia), LUS alone does not configure a safe and reliable method for discharging patients and referring the follow-up at home.

In the present study, LUS showed a low specificity (62.5%). This result is even higher than that reported by a recent meta-analysis by Cochrane on thoracic imaging tests for the diagnosis of COVID-19, calculating for LUS a specificity of 45% (32). Indeed, ultrasound findings in COVID-19 pneumonia are not specific. In fact, the most frequently advocated as typical COVID-19 LUS signs (i.e., a thickened and irregular pleural line with an increased number of B-lines) have been reported by literature in several pathological conditions, ranging from lung fluid accumulation [i.e., heart failure (33) or end-stage renal disease accompanied by pulmonary congestion (34)], lung injury and/or inflammation [i.e., acute respiratory distress syndrome (35), other viral or bacterial pneumonia (36), pulmonary contusion (37), acute exacerbation of chronic obstructive pulmonary disease (COPD) and emphysema (38), acute bronchial asthma (39, 40), neoplastic lymphangitis (41)], till to interstitial remodeling [i.e,. bronchiectasis (42), pulmonary fibrosis (16, 43–45)]. Similar alterations can occur even in healthy individuals and should be interpreted in relation with age (46). Small subpleural nodulations interrupting pleural line's continuity have been described in acute respiratory distress syndrome (35), other viral pneumonia (36), tuberculosis (16), pulmonary fibrosis (16, 45), and neoplasms (41). Moreover, subpleural consolidations may be visible also in other viral and non-viral pneumonia, atelectasis and lung cancer (47). Thus, the not specific echographic pattern of consolidations cannot allow to distinguish one condition from another.

In addition, overlapping conditions may be present in COVID-19 patients, especially in more severe cases (16, 48). Unlike to other studies, we enrolled every adult patient with confirmed SARS-CoV-2 infection referred to our COVID-19 center, without excluding patients with known chronic cardiac and pulmonary comorbidities. Therefore, we can assume that our cohort of consecutive patients is representative of a real-life setting. Moreover, in the present study we recorded any collateral findings at admission Chest CT indicative of overlapping conditions. They included signs of heart failure, lung fibrosis, bronchiectasis and subpleural emphysema. As expected, their frequency increased with the severity of disease. Furthermore, among those with severe radiological disease, five patients had an overlapping bacterial pneumonia. Given the low specificity of LUS findings, we were unable to discern COVID-19 alterations from such pre-existing or supervening conditions. Although in cases of interstitial lung remodeling the echographic pattern is practically identical to that of COVID-19 pneumonia, in heart failure the hyperechoic pleural line is generally thickened but more blurred and less fragmented. However, these subtle differences cannot be easily recognized, particularly by an inexperienced eye. In the remaining cases with no pulmonary involvement detectable on CT scan, a “false-positive” LUS was most likely attributed to the following overlapping conditions: chronic heart disease (six cases), bronchiectasis (two cases), and paraseptal emphysema (one case). Given these bases, a positive RT-PCR testing is always required to confirm the diagnosis. The risk of a diagnosis based on the LUS examination is the admission of patients with any other disease, but without SARS-Cov-2 infection, among COVID-19 patients.

In our study population, given a prevalence for COVID-19 pneumonia at chest CT of 90.77%, the positive predictive value of LUS was of 93.7%. Despite LUS findings may show good positive predictive values in the context of COVID-19 pandemic (i.e., high “*a priori*” probability of disease in the presence of respiratory symptoms), the ability of LUS to rule out COVID-19 pneumonia in normal condition may become far from sufficient as the prevalence of COVID-19 decreases (e.g., non-epidemic setting) and/or the prevalence of diseases producing similar findings (e.g., ILD, influenza, bacterial pneumonia, and heart failure) increases. An AUC value of 0.596 confirmed the poor performance of LUS in discriminating COVID-19 pneumonia with respect to Chest CT.

Finally, LUS examination has the disadvantages to be strongly operator-dependent and generally based on subjective observations (47, 49, 50). The thickness and appearance as well as the number of B-lines of hyperechoic pleural line may vary basing on the type of probe used (e.g., low-frequency convex probe or high-definition linear transducer), the angle of incidence of the probe, the ultrasound scan (e.g., longitudinal, transverse, or oblique), and the operator's experience (47, 51). The simple change of positioning of the probe with respect to the curvature of the patient's chest and the patient's respiratory rate may increase the perceived occurrence of such artifacts (15, 51, 52). With these considerations in mind, it is clear that the risk that LUS will be ineffective in untrained hands may be more harmful than helpful.

## Conclusions

In our study, LUS showed low sensitivity (high number of false negatives) and low specificity (high number of false positives) in assessing signs of COVID-19 pneumonia. Given these performances, compared with chest CT scan, LUS carries from one hand the risk of underdiagnosis and/or underestimation of the extent of the disease and, from the other hand, the possibility to erroneously classify pre-existing or overlapping conditions as COVID-19 pneumonia. In the setting of COVID-19 pandemic and by trained hands, LUS may represent an expanded clinical evaluation to suggest even the presence of “a virosis,” when integrated into a multimodal approach including clinical, epidemiological, laboratory, and radiologic findings (53, 54). In any case, viral testing confirmation is always required.

## Data Availability Statement

The raw data supporting the conclusions of this article will be made available by the authors, without undue reservation.

## Ethics Statement

The studies involving human participants were reviewed and approved by Ethical Commitee of Fondazione Casa Sollievo della Sofferenza, San Giovanni Rotondo, Italy. The patients/participants provided their written informed consent to participate in this study.

## The CSS-COVID-19 Group

Paolo E. Alboini, Annibale Antonioni, Filippo Aucella, Giovanni Battista Bochicchio, Cristiano Carbonelli, Massimo Carella, Giulia Castorani, Marco Castori, Antonella Centonze, Gianluca L. Ciliberti, Massimiliano Copetti, Michele Corritore, Leonardo D'Aloiso, Maria M. D'Errico, Angela de Matthaeis, Alfredo Del Gaudio, Annabella Di Giorgio, Lazzaro Di Mauro, Lucia Florio, Andrea Fontana, Vincenzo Giambra, Elvira Grandone, Vincenzo Inchingolo, Michele Inglese, Maria Labonia, Antonella La Marca, Tiziana Latiano, Maurizio Leone, Evaristo Maiello, Alessandra Mangia, Carmen Marciano, Simonetta Massafra, Giuseppe Miscio, Grazia Vittoria Orciulo, Nicola Palladino, Rita Perna, Pamela Piscitelli, Matteo Piemontese, Michele A. Prencipe, Pamela Raggi, Maria Grazia Rodriquenz, Daniele Sancarlo, Vincenzo Trischitta, Michele Zarrelli, Pasquale Vaira, Doriana Vergara, and Angelo Vescovi.

## Author Contributions

CMIQ, MS, and AM contributed to the conception and design of the study, to acquisition and interpretation of data, and to writing the manuscript and revise it critically. MM, CB, RR, DL, MF, GS, PT, GR, AS, BF, SD, VM, and AG contributed to acquisition and interpretation of data. All authors contributed to manuscript revision, read, and approved the submitted version.

## Conflict of Interest

The authors declare that the research was conducted in the absence of any commercial or financial relationships that could be construed as a potential conflict of interest.
